# Enhancing Cartilage Repair: Surgical Approaches, Orthobiologics, and the Promise of Exosomes

**DOI:** 10.3390/life14091149

**Published:** 2024-09-11

**Authors:** Jacob Singer, Noah Knezic, Jonathan Layne, Greta Gohring, Jeff Christiansen, Ben Rothrauff, Johnny Huard

**Affiliations:** Linda and Mitch Hart Regenerative and Personalized Medicine, Steadman Philippon Research Institute, Vail, CO 81657, USA; jsinger@sprivail.org (J.S.); nknezic@sprivail.org (N.K.); ggohring@sprivail.org (G.G.); jchristiansen@thesteadmanclinic.com (J.C.); ben.rothrauff@gmail.com (B.R.)

**Keywords:** articular cartilage, osteoarthitis, orthobiologics, exosomes, microfracture, chondrocytes

## Abstract

Treating cartilage damage is challenging as its ability for self-regeneration is limited. Left untreated, it can progress to osteoarthritis (OA), a joint disorder characterized by the deterioration of articular cartilage and other joint tissues. Surgical options, such as microfracture and cell/tissue transplantation, have shown promise as techniques to harness the body’s endogenous regenerative capabilities to promote cartilage repair. Nonetheless, these techniques have been scrutinized due to reported inconsistencies in long-term outcomes and the tendency for the defects to regenerate as fibrocartilage instead of the smooth hyaline cartilage native to joint surfaces. Orthobiologics are medical therapies that utilize biologically derived substances to augment musculoskeletal healing. These treatments are rising in popularity because of their potential to enhance surgical standards of care. More recent developments in orthobiologics have focused on the role of exosomes in articular cartilage repair. Exosomes are nano-sized extracellular vesicles containing cargo such as proteins, lipids, and nucleic acids, and are known to facilitate intercellular communication, though their regenerative potential still needs to be fully understood. This review aims to demonstrate the advancements in cartilage regeneration, highlight surgical and biological treatment options, and discuss the recent strides in understanding the precise mechanisms of action involved.

## 1. Introduction

Cartilage is a complex tissue characterized by its strong and flexible properties, playing a key role in protecting joints from wear and tear. During embryologic development, stem cells from the mesoderm progeny undergo chondrogenesis, forming chondrocytes that produce a high concentration of extracellular matrix (ECM) [1,2]. This highly specialized matrix consists of fibrous tissue and various combinations of proteoglycans and glycosaminoglycans. There are two main subsets of cartilage: hyaline and fibrocartilage. Hyaline cartilage is the most abundant type of cartilage in the human body and resides in specific joints such as articular cartilage (AC). Composed primarily of type II collagen, it can resist compressive forces and reduce friction [3]. By nature, AC is predominantly avascular and acellular; composed mainly of secreted ECM, this tissue is optimal for handling the mechanical stresses of the joint. However, these characteristics make repairing damaged cartilage difficult [4,5]. When AC is damaged, it has a limited ability to regenerate autonomously due to low numbers of dividing cells and inefficient recruitment of cellular repair mechanisms resulting from a lack of vascularization that limits what is possible in native repair [4]. If left untreated, cartilage damage can become widespread and lead to diseases like osteoarthritis (OA). As such, there is a collective effort in orthopedics to reconstruct or regenerate articular hyaline cartilage to treat acute injury and delay the development of osteoarthritis.

While current interventions have shown effectiveness in managing the symptoms of cartilage damage, such as pain medications, anti-inflammatory injections, and surgical procedures, they have their limitations. These methods have succeeded in improving function and halting further degeneration, but achieving complete restoration of functional AC remains challenging [6]. Instead, the formation of mechanically inferior fibrocartilage is an expected outcome, contrasting with the desired hyaline cartilage native to joints. This is where the field of regenerative medicine comes in, aiming to overcome these limitations by harnessing the body’s innate healing capabilities.

This review will delve into the most prevalent therapeutics for articular cartilage injury and highlight promising developments in regenerative medicine. It will specifically explore how orthobiologics and the emerging therapeutic potential of exosomes have advanced our current understanding of cartilage repair and its unique anatomy that is challenging to regenerate. Exosomes present a unique opportunity as a bioactive molecule to modulate chondrogenic cellular processes in cartilage damage to actively communicate with neighboring chondrogenic cells to heal in a controlled and specified manner. The following sections will cover the current research and findings in cartilage damage in orthopedic conditions and discuss the future directions in translating orthobiologics and exosome-based therapies from bench to bedside. 

## 2. Cartilage Overview

This section will cover the structure, composition, and types of human cartilage essential for maintaining healthy joints, further elaborating on damage to cartilage and its potential to lead to osteoarthritis (OA). 

### 2.1. Human Cartilage: An Overview

Cartilage is a strong, flexible, non-vascular connective tissue. There are three types of cartilage with unique roles throughout the body: (1) hyaline cartilage (HC)—the most abundant form, has a glassy appearance and is found in joints at the ends of bones, the trachea and parts of the skull; (2) fibrocartilage (FC)—a solid and fibrous tissue, appears opaquely white and is found predominantly in the intervertebral disks of the spine and at osteotendinous/osteoligamentous junctions; and (3) elastic cartilage (EC)—a yellowish, pliable, non-load-bearing tissue found in the epiglottis, the external ear, and the auditory tube of the middle ear [7]. Each cartilage type has a low density of cells in structured extracellular matrices, the unique architectural components of which are vital for proper cartilage growth and function [8]. Most diseases of cartilage, including OA, involve significant, detrimental changes in the organization of their cartilaginous ECM due to contributing factors such as joint injury [8,9,10].

### 2.2. Articular Cartilage: Lessons from Early Chondrogenesis 

AC is composed of hyaline cartilage found on bone surfaces within synovial joints or diarthroses. Synovial joints are contained by fibrous joint capsules that are continuous with the periosteum of each articulating bone and filled with synovial fluid [11]. Cartilage cells (chondrocytes) found in AC experience constant shifts in the joint’s physicochemical environment, receiving various mechano-chemical signals. The ECM secreted by AC chondrocytes is a unique blend of primarily type II collagen molecules, proteoglycans, and glycoproteins [7,12]. However, like other cartilage types, AC has minimal self-restoring properties when compared with bone, and defects frequently persist without healing [12,13,14]. 

The inability of AC to spontaneously heal presents a significant challenge, not only to surgeons and clinicians attempting to help patients recover from cartilage injury/degradation, but also to scientists searching for a reliable approach to regenerate AC. The only example of spontaneous AC formation in vivo is during the early stages of embryonic development [15]. Despite the challenges, significant advances in our understanding of AC chondrogenesis have highlighted key genetic, cellular, and molecular contributors, some of which are targets for new or improved regenerative therapies. 

The process of AC chondrogenesis begins when mesenchymal stromal/progenitor cells (MSCs) from the mesoderm start to condense at the ends of bones, eventually committing to the chondrogenic lineage [16]. These MSCs differentiate into chondroblasts and chondrocytes, which begin secreting cartilage matrix, a behavior initially shown to correlate with intracellular collagen gene expression [17,18]. During MSC aggregation and throughout the maturation of chondrocytes and the ECM, chondrogenesis is controlled by a complex orchestra of genetic, molecular, and cellular signals, including balanced stimulation from the transforming growth factor-beta (TGF-ß) and bone morphogenic protein (BMP) families (Table 1) [19]. The expression of Sox9, the master regulator of the chondrocyte lineage, can be directly induced by BMP signaling, as demonstrated in vivo using animals with null mutations in Sox9. Animals with homozygous null mutations in Sox9 did not form cartilage, and those with heterozygous mutations exhibited defects in all cartilage primordia [16].

### 2.3. Cartilage Defects and Osteoarthritis

Focal cartilage injury is common in active patients. Typically, these injuries result in isolated damage and can be a result of high impact, impingement, fracture, surgical operation, or ligament tear. Focal injuries require treatment to reestablish quality of life and limit further systemic degradation to the joint [31]. OA correlates to previous joint trauma factors such as inflammation, joint abnormalities, avascular necrosis, and other conditions can lead to joint malformities, as well as cartilage degradation that commonly leads to the progression of OA [31].

**Figure 1:**
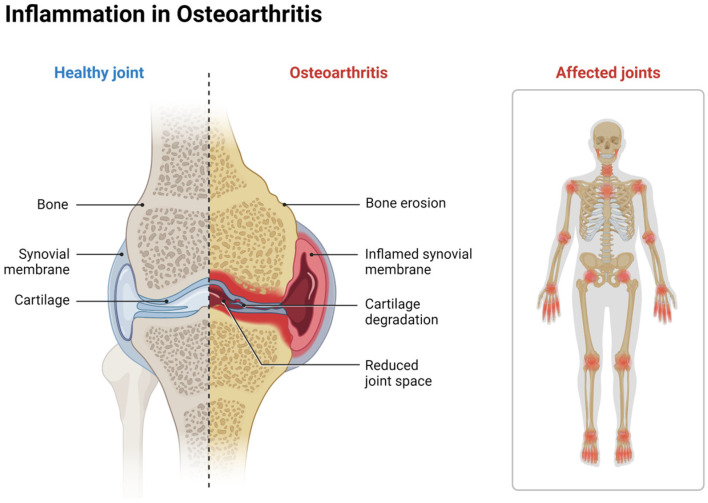
Osteoarthritis is the most common joint disorder that is associated with reduced joint space, inflammation, and cartilage degradation. Created with Biorender.

OA is the most common joint disorder in the world and a significant burden on individuals and society. In 2019, The Lancet’s Global Burden of Disease study (GBD) reported 528 million people suffering from this condition, an increase of 113% since 1990 [32]. Caring for patients with OA is often costly to individuals, families, and healthcare systems. In 2017, total direct healthcare spending associated with OA reached USD 41.7 billion in the United States alone [33,34]. Despite high spending, there is still no known cure for OA, and many existing treatments seemingly target symptom management only. OA causes irreversible cartilage and subchondral bone degeneration, joint space narrowing, subchondral bone thickening (or sclerosis), bone spur (or osteophyte) formation, and painful loss of joint function over time. Due to the lack of a neural network in the joint space, cartilage damage can go unnoticed during the progression to OA; patients are usually asymptomatic until later stages, and diagnosis may be difficult until after irreparable joint damage has occurred [35]. Post-traumatic OA (PTOA) occurs when the onset of OA is triggered by joint trauma, which primarily affects the knee and ankle joints and accounts for about 12% of all OA cases [36,37]. Approximately 50% of all patients who suffer from traumatic joint injury (e.g., anterior cruciate ligament tears) will experience PTOA, though it may take 10–15 years to reach full development [36]. While the exact pathogenesis of OA is not understood, the current consensus suggests a complex interplay of dysfunctional cell behaviors, imbalanced metabolic homeostasis in the chondral/subchondral layers of bone ends, and significant inflammation and fibrosis in the surrounding joint (Figure 1) [38,39,40]. 

Current best practices for preventing and treating the cartilage degeneration seen in OA vary based on etiology and location, though generally, emphasis is placed foremost on maintaining joint health throughout life by engaging in regular exercise, avoiding joint overuse, making good nutrition choices, weight management, and early intervention when injuries occur. Few pharmacologic interventions for OA exist, however, anti-inflammatory medication and intra-articular injections are used for temporary pain relief [41]. For patients with highly active lifestyles—professional athletes, weekend warriors, or manual laborers who regularly experience intense joint strain—orthopedic and sports/occupational medicine specialists focus on optimizing joint biomechanics to restore adequate function. Non-surgical methods that cater to improving biomechanics may include targeted exercise programs or tailored physical therapy. Though some may find relief with these interventions, many seek out surgical treatment due to OA’s degrading nature. Various surgical techniques have differing levels of success, depending on the initial injury or condition and, in some cases, emerging biological approaches have been used [42]. These techniques include cartilage resurfacing, membrane-induced autologous chondrocyte implantation (MACI), microfracture, osteochondral allografting, and the osteoarticular transfer system (OATS) procedure. However, some surgical and regenerative techniques used to stimulate the biological process of cartilage formation have come under scrutiny due to mixed results from long-term follow-ups. Despite the brief pain relief experienced by patients under current OA management practices, many will eventually proceed with costly joint-replacement surgery (arthroplasty) to overcome progressive pain and disability. 

### 2.4. Tissue Response to Cartilage Damage

**Figure 2:**
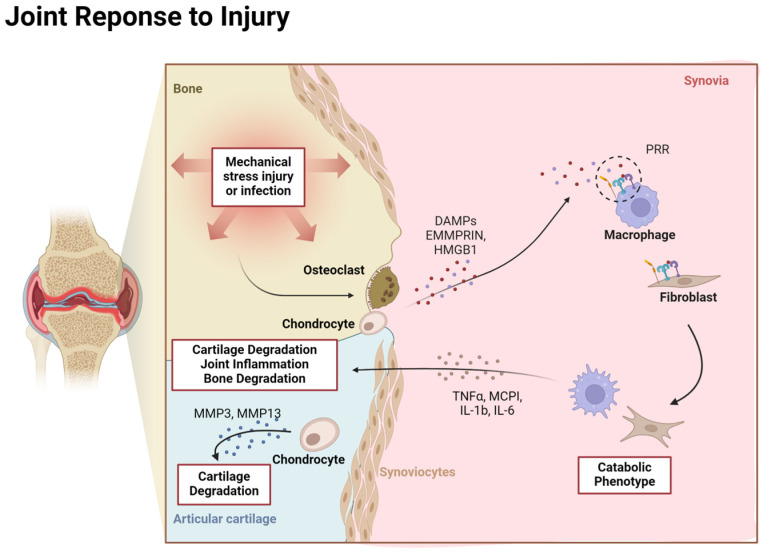
A mechanical stress injury or infection to the joint triggers the osteoclast and damaged chondrocytes to release alarmins into the synovia (DAMPs, EMMPRIN, HMGB1). Alarmins then bind to pattern-recognition receptors (PRR) on synovial cells, which then release pro-inflammatory mediators (TNFa, MCPI, IL-6). Amplification of inflammation within the synovia causes chondrocytes to release degrading factors (MMP3, MMP13) that can lead to further cartilage degradation, joint inflammation, and bone remodeling. Created with Biorender.

Injury to AC and subchondral bone triggers several biological responses. Similar to bone cells, chondrocytes have mechanosensory receptors on their surface membrane, which allows them to react to shear stress, compression, or tension (Figure 2). The mechanic force on the membrane triggers intracellular signaling pathways, such as MAPK, Wnt/b-catenin, and Indian Hedgehog, that induce upregulation of genes involved in ECM production [43]. Furthermore, individually damaged chondrocytes may release damage-associated molecular patterns (DAMPs), such as fibronectin fragments, extracellular matrix metalloproteinase inducer (EMMPRIN), and high mobility group box 1 (HMGB1) [44]. These molecules interact with pattern recognition receptors (PRRs) like toll-like receptors (TLRs) on chondrocytes and other synovial cells to initiate repair pathways that trigger pro-inflammatory cytokine/chemokine release [45,46]. Undamaged, nearby chondrocytes may also augment their metabolic activity due to the influence of inflammatory cytokines such as IL-1b, TNF-α, monocyte chemoattractant protein-1 (MCP1), and Interleukin-6 (IL-6), which are often present in the synovial fluid following acute injury [47,48]. These mediators amplify the inflammatory response, stimulating chondrocytes to release matrix metalloproteinases (MMPs), like MMP3 and MMP13, which degrade the cartilage matrix into collagen or proteoglycan fragments that may also behave as pro-inflammatory agents (Figure 2) [49]. This degradation is typically tightly regulated by tissue inhibitors of metalloproteinases (TIMPs). Some ECM matrix fragments like collagen peptides and hyaluronan oligosaccharides can interact with cell surface receptors like CD44 and integrins, activating inflammatory signaling pathways such as NF-κB and leading to further production of catabolic cytokines and further enzymatic activity [50]. 

Despite the known healing benefits from acute inflammation in many injury states, synovial joints appear to lose the regenerative capacity for hyaline cartilage formation, generating fibrocartilage within defects [51]. Fibrocartilage, with a matrix rich in type I collagen, has been demonstrated to form more quickly than hyaline cartilage. The formation occurs because the type I collagen gene expression is less sensitive to the local environment than type II collagen expression, and readily forms even during acute inflammation [52]. Type II collagen, which is only upregulated in high enough concentrations for hyaline cartilage formation when the microenvironment is optimal for chondrogenesis, relies on specific growth factors and the absence of inflammatory cytokines [53]. In the setting of joint injury, the environment often does not adhere to the strict needs for chondrogenesis. The environment is particularly important when the zonal organization of the cartilage, critical for its normal function, is disrupted [54,55]. Hyaline cartilage exhibits a unique architecture from the superficial to the deep layers, with chondrocytes and the ECM organized to enable the tissue to withstand its mechanical demands [51,56,57]. This structural organization is difficult to replicate during the repair process, and the resultant fibrocartilage lacks the finely tuned mechanical properties of native hyaline cartilage [54,58,59].

Emerging regenerative medicine strategies aim to recreate the microenvironment necessary for hyaline cartilage formation. Techniques such as scaffold-based tissue engineering, autologous chondrocyte implantation (ACI), and the application of bioactive molecules encapsulated in biomaterials are developing to provide the cues necessary for MSCs to differentiate into chondrocytes capable of synthesizing a hyaline-like cartilage matrix [60,61]. These strategies mimic the embryonic development of articular cartilage, wherein a finely tuned cocktail of growth factors, mechanical cues, and cell–matrix interactions guide the formation of the functional zonal architecture seen in native hyaline cartilage.

## 3. Cartilage Repair Techniques

AC deterioration can be accelerated through sports injury, trauma, natural aging, and comorbidity. Current treatment options for AC damage only address pain and the consequences of deterioration since no procedure or treatment has yet to rejuvenate the joint and re-establish native function [62]. Existing treatments include corticosteroid injections, anti-inflammatory medications, and surgical interventions to debride painful osteophytes or replace the joint altogether [63,64]. 

The ideal treatment(s) for damaged cartilage would not only prevent or at least mitigate the metabolic changes that damaged chondrocytes undergo, but also recapitulate the balance of anabolic and catabolic events necessary for tissue homeostasis, and promote chondrogenic progenitor cells homing to the defect [65]. The potential of future research in this area is promising, as it could lead to innovative approaches that circumvent native repair shortcomings by sourcing the materials necessary for the body to complete healing of the defect, often delivering them locally. The remainder of this chapter will discuss the current standards of intervention and repair for AC damage, highlighting the pros and cons of various operative/non-operative regenerative techniques, and proposing these exciting future directions for research.

### 3.1. Surgical Approaches for Cartilage Repair 

#### 3.1.1. Microfracture or Bone Marrow Stimulation

Bone marrow stimulation through microfracture triggers the body’s natural healing response to promote cartilage defect repair [66]. Several standard arthroscopic procedures aim to trigger this response by establishing blood flow, allowing the recruitment of biologic materials and mechanisms to fill in the defect. The following standard methods have shown efficacy in symptom reduction and return to function, especially when the defect is minor. 

Microfracture surgery, initially developed by Dr. Richard Steadman in the early 1980s, is a procedure to make small perforations in the subchondral bone perpendicular to the surface in full-thickness chondral defects ranging less than 2–3 cm in size [66,67]. A specialized awl creates perforations spaced 3–4 mm apart and reaches a specific 2–4 mm depth to release the crucial elements in bone marrow [68]. A microfracture is usually completed with another type of joint surgery to restore total joint functionality. Bone marrow contains mesenchymal stem cells, growth factors, and other healing factors such as platelets. Penetrating the subchondral bone allows for the promotion of a marrow clot to form at the base of the chondral lesion, providing an optimal environment for progenitor stem cells to differentiate into chondrocytes and undergo chondrogenesis. However, recent evidence has shown that bone marrow stimulation creates an environment that promotes the formation of fibrocartilage rather than the hyaline cartilage native to the synovial joints in which microfracture is commonly performed [69]. In some respects, stem cell differentiation bypasses distinct mechanisms important for chondrogenesis and the promotion of type II collagen; instead, it forms type I collagen that defies the natural niche of the synovial joint [70]. Due to this, creating an environment specific for chondrogenic production leading to hyaline cartilage is of utmost importance because the creation of cartilage not native to the site of injury could be a cause of failed surgical intervention, leading to revision cases and poor clinical outcomes. It is essential to replicate the native niche where chondrogenesis and aggrecans can perform to their full potential. 

#### 3.1.2. Cartilage Resurfacing

Resurfacing is often a necessary step in surgical procedures for significant defects [66,71]. The benefit of these procedures is that hyaline cartilage can be restored to the defect area, which is critical for more extensive defects where the inferior mechanics of fibrocartilage may play a more noticeable role. The downside to resurfacing surgeries is that they are often more involved and performed through open surgery rather than arthroscopically. 

Matrix-induced autologous chondrocyte implantation (MACI) is a two-step procedure in which a small amount of healthy cartilage is removed from a non-weight-bearing area in the patient and sent to a lab where it is grown on a collagen matrix [4]. A second procedure is required to place the newly grown cartilage graft into the patient’s cartilage defect. MACI treatment is notably performed in chondral surface defects as it is not an ideal option in treating injuries involving the subchondral bone [4]. In addition, having to grow the new graft for one month ex vivo and then undergo additional surgery to implant the graft can lead to more complications.

Osteochondral Autograft Transplantation (OAT) is a procedure that treats articular cartilage repair when there is damage to the subchondral bone as well [72,73]. The method begins by harvesting a healthy cartilage graft from the patient’s knee from a non-weight-bearing area, then removing the damaged area of cartilage, creating a hole or plug for the insertion of the graft. The cylinder-shaped graft is then matched to the surface of the defect and pushed into place, leaving a smooth articular surface. However, when defects are too significant or widespread, it is challenging to treat autologously as there is only so much preserved cartilage to remove and transplant. Osteochondral allograft transplantation (OCA) is a procedure similar to the traditional OAT method. However, instead of using the patient’s tissue, the graft is taken from a cadaver tissue donor, which provides an option to treat more complex chondral defects [73]. Its limitation is that the tissue is not native to the patient, which can introduce a new environment to the knee joint, limiting its native potential. 

### 3.2. Orthobiologics for Cartilage Repair

#### 3.2.1. Current Orthobiologic Treatments

With the Food and Drug Administration (FDA) regulating the US utilization of more advanced/manipulated stem cell technologies, orthobiologics have emerged as therapeutic agents with the ability to promote tissue repair by recruiting pro- and anti-inflammatory responses, inducing faster healing times for soft tissue injuries [74,75]. Orthobiologics harness and enhance the patient’s natural healing properties and involve minimally manipulated and less invasive procedures, such as injections or the implantation of biological products. Orthobiologics have the potential to reduce pain and inflammation without surgical complications as well. Currently, available treatment options include platelet-rich plasma (PRP), bone marrow aspirate concentrate (BMAC), and micronized allogenic cartilage matrix (MCM) [76,77].

**Figure 3:**
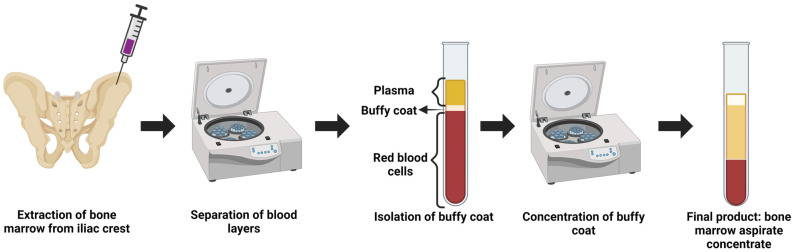
Depiction of the extraction of BMA from the iliac crest. BMA is centrifuged to separate the specific blood marrow components and concentrated to BMA. Created with Biorender.

PRP is an autologous blood product where patients’ peripheral blood is drawn and processed to capture and concentrate the platelets, which are then resuspended in plasma [78,79]. Through centrifugation at a specific speed, usually 1500 g at 10 min, blood can be separated into three distinct layers: plasma (55% composition), the “buffy coat” (1% composition), and red blood cells (RBCs) (45% composition) [80]. Platelets reside in the “buffy coat”, a white-colored layer atop the RBCs containing platelets, mononuclear cells, and white blood cells (WBCs). This concentrate can then be prepared for injection into a specific site of injury that may promote pro- and anti-inflammatory reactions that induce innate healing by releasing essential growth factors, such as TGF-β, PDGF-α, and VEGF. The release of these biologically active molecules, platelets, and specific clotting factors promotes a cascade of events leading to a hemostatic plug formation at the injury site. This formation leads to a fibrin mesh, allowing tissue growth. Expanding upon the biologic affects from peripheral blood as a source for autologous orthobiologics, bone marrow consists of fat and immature blood-forming stem cells containing mesenchymal stromal cells (MSCs) that promote repair and regeneration [81]. Bone marrow aspirate concentrate (BMAC) involves a minimally invasive procedure where bone marrow aspirate (BMA) extraction occurs generally from the iliac crest (Figure 3) [82]. BMA is then concentrated, capturing the “buffy coat” containing the essential growth factors and stem cells circulating in the bone marrow [83]. Reducing the number of erythrocytes and leukocytes is important while increasing the concentration of the mononuclear region and hematopoietic stem cells [84,85]. BMAC is unique as it is an autologous product that can be retrieved through a minimally invasive procedure at a reasonable price, considering the market for stem cell therapy. Recent evidence shows that BMAC can augment cartilage repair and form more native articular cartilage [85,86]. However, BMAC only contains a small percentage of MSCs as they only make up 0.01–0.001% of the total nucleated cell population [87]. It is necessary to explore more optimal ways to retrieve BMAC and how lesser restrictions on cellular manipulation may increase the number and quality of MSCs captured in BMAC, which in return may lead to even better quality cartilage repair. 

An additional modality of utilizing orthobiologics in the clinic space involves the use of a matrix to further mimic native environments for healing. Micronized Allogenic Cartilage Matrix (MCM) is dehydrated and decellularized cartilage that contains the native components of articular cartilage [88]. Recent evidence has shown that combining MCM with orthobiologics can synergistically lead to more native cartilage formation [89]. The scaffold-like template provides a support for the formation of hyaline cartilage and act as a delivery system for other therapies, such as PRP and stem cells [90,91]. Recent studies have shown the promise of using MCM as a scaffold for MSCs, promoting an environment suitable for MSCs to release bioactive factors that are essential for promoting cell migration and vascularization [92,93]. MCM provides MSCs with the appropriate structure for optimal cell viability, showing that rehydrating the MCM can lead to nearly 100% cell viability [88]. This platform has demonstrated the potential of maximizing the body’s incorporation of viable cartilage and loading systems for important biologic therapies like BMAC and PRP.

#### 3.2.2. Limitations of Orthobiologics

Orthobiologics present a novel opportunity to use the body’s natural resources to initiate the innate healing response [33]. Since they are injected into the injury site to promote direct healing to the site needed for recovery, this targeted communication creates a fast-acting mechanism for cell-to-cell interaction. The efficacy of orthobiologics largely depends on what the therapy application is, as it is well known for its ability to help relieve inflammation and reduce pain. When combined with surgery, the success rate increases, however, it is still worth noting the quality of the orthobiologic products and the state of the native tissue after injury [94,95]. As an autologous product, the healing capacity of orthobiologics is limited to the patient’s age, extent of cartilage damage, and the specific type of orthobiologics used. Maximizing the concentration of MSCs in a reduced volume allows the therapeutic delivery of the cellular concentrate, secretome, and extracellular vesicles to a site of orthopedic injury or surgical repair. One limiting factor in BMAC is that the MSCs comprise only a tiny fraction of the total nucleated cell population. There is scientific debate about whether the small population of MSCs promotes the repair process or if the MSC derived extracellular vesicles that promote the healing process. 

### 3.3. Microfracture Enhancement Therapy 

Another emerging method to overcome current treatment disadvantages, specifically those seen in microfracture, is microfracture enhancement therapy. New research has shown that combining specific drugs with the procedure can lead to higher-quality cartilage, with higher concentrations of type II collagen representing hyaline cartilage (Table 2). 

Losartan is a common drug used to treat high blood pressure and has recently shown evidence that, when given at a low dose, can block TGF-β1. The downregulation of TGF-β1 binding to fibroblast TGF-β receptors deactivates the pro-fibrotic response [99]. Recent evidence has shown that oral Losartan (10 mg/kg/day) improved microfracture-mediated cartilage repair and increased hyaline cartilage production in a rabbit osteochondral defect model [21]. Furthermore, intra-articular Losartan (1 mg/knee) has significantly improved microfracture-mediated cartilage repair [100,101]. These results have led to several clinical trials using Losartan to treat cartilage injury and disease [5,102,103]. 

Angiogenesis is also directly linked to inflammation and, when uncontrolled in the cartilage repair system, can lead to chondrocyte hypertrophy and endochondral ossification. These changes directly create an environment unsuitable for native tissue formation and homeostasis. Blocking angiogenesis has recently shown promise in treating cartilage defects through Avastin. This standard cancer treatment drug has shown promise in blocking angiogenesis by inhibiting catabolic reactions while stimulating anabolic functions [104,105]. Systemic administration of Avastin can improve microfracture-mediated cartilage repair [106]. More recently, Avastin has shown promise as an anti-VEGF therapeutic, directly inhibiting VEGF’s role in inhibiting the anabolic function of articular chondrocytes, leading to more production of chondrocytes, subsequently forming type II collagen. Intra-articular injection of FDA-approved Avastin (VEGF antibody) also enhanced microfracture-mediated cartilage repair by increasing hyaline cartilage [28,97,106,107]. Since cartilage repair depends on chondrogenic signaling to promote subchondral bone cells to differentiate into chondrocytes, the use of chondrogenic growth factor shows more significant differentiation potential. Delivery of BMP7 or BMP2 using different scaffolds shows hyaline cartilage regeneration [108,109]. However, it is important to note that further research is needed in this area to understand and fully optimize these methods. 

Another issue that plagues individuals with cartilage repair is the accumulation of senescent cells at the injury site. Senescence is when cells reach a stable state of cell-cycle arrest while remaining metabolically active. During this state, senescent cells respond to molecular or cellular damage by initiating the release of various growth factors and cytokines, making up the senescence-associated secretory phenotype (SASP) [110]. Cellular senescence acts as a self-defense mechanism as it is known to arrest the proliferation of damaged cells. However, this mechanism creates an environment where the cells resist apoptosis while accumulating at the injury site, unable to be cleared out [111]. Cartilage damage has led to recent research indicating a chondrocyte SASP pathway that includes the upregulation of MMP-1 and MMP-13, known for its role in cartilage degradation and in the progression of OA [112,113,114]. Fisetin, a natural over-the-counter supplement, has shown remarkable promise as a senolytic drug that can promote apoptosis in senescent cells by turning off the pro-survival pathway, leading to the elimination of senescent cells [115,116]. As a polyphenol and flavonoid with antioxidants, fisetin has an anti-inflammatory and senolytic effect shown to improve microfracture-treated cartilage repair using osteochondral defect model animals [98,117]. Recent studies have shown the exciting potential of the combinatorial effect of fisetin and BMAC on cartilage repair, and the enhancement of microfracture. The synergy between the two created an environment to produce higher-quality hyaline cartilage with more robust mechanical and structural properties [117].

### 3.4. Exosomes, an Emerging Therapeutic for Cartilage Repair

#### 3.4.1. Introduction to Extracellular Vesicles

**Figure 4:**
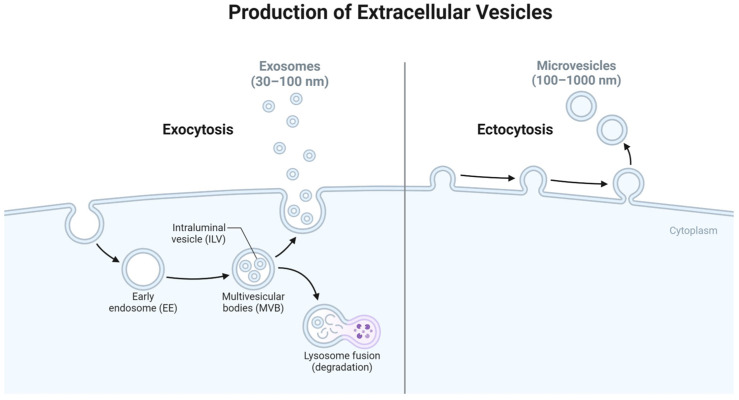
The biogenesis of extracellular vesicles (EVs)—exosomes are the smallest EVs, originating from the endosomal pathway of the signaling cell and released through exocytosis. Larger microvesicles’ origins differ from those of their smaller counterparts as their cargo comes directly from the cytoplasm of the signaling cell and is released through ectocytosis. Created with Biorender.

As discussed, the microenvironment of the joint space has a profound impact on the repair and regeneration of AC. Orthobiologics present a promising future for restoring AC damage, and exosomes are at the forefront due to their lack of immunoreactivity and simplicity as a result of their acellular nature [118,119,120]. Exosomes play a critical role in cellular communication, the transmission of materials, and cellular machinery [121,122]. Extracellular vesicles (EVs) are nanometer-sized lipid molecules that facilitate cell-to-cell communication and are present throughout the body [123,124,125]. They play a role in both stimulating and suppressing cellular responses through delivery of bioactive cargo derived from the signaling cell. EVs deliver their cargo systemically to cells through various targeting mechanisms. EVs present a unique opportunity to facilitate and enhance the intercommunication between impaired tissues and introduced therapeutic agents [126]. EVs’ ability to fuse with the plasma membrane through exocytosis and ectocytosis presents an opportunity to potentiate the reparative process and ensure vital cues are robustly transmitted and executed within biologic pathways (Figure 4). Tian et al. used rat pheochromocytoma (PC12) cell-derived exosomes to study the endocytosis pathway by live cell microscopy, revealing the PC12 cells were internalized and carried to the perinuclear region where they can directly fuse with lysosomes or the plasma membrane [127,128]. 

#### 3.4.2. Origin of Exosomes 

Exosomes are a type of extracellular vesicle that are characterized by their size of 30–150 nm in diameter and their endosomal origin (Figure 4) [129,130]. Exosomes originate from a variety of cells and include a variety of exosomal cargo including signaling proteins, microRNA (miRNA), and messenger RNA (mRNA) invaginated from cytoplasmic space [131]. Exosome production begins in endosomal specific organelles in which cytoplasmic cargo is sorted by inward budding of the membrane, forming intraluminal vesicles (ILVs) [132,133]. The formation of the ILVs are the precursors to multivesicular bodies (MVBs) that are either degraded by the lysosome or fused with the plasma membrane to release the sorted vesicles or exosomes [134].

#### 3.4.3. Composition

Exosomes have a unique composition distinguishing them from other extracellular vesicles. Due to their biogenesis through the endosomal systems complex sorting, exosomes represent a more homogenous type of extracellular vesicle [135]. Comprised of a lipid bilayer different than that of the signaling cell, this distinct factor creates the optimal cargo transporter as it can easily pass through biologic barriers and be targeted for uptake by cells [136]. Distinctive surface markers integrated with the lipid bilayer unique to exosomes, such as CD9, CD63, and CD81, act as common biomarkers for identification and purification. Additionally, these surface markers, along with proteins specific for cellular signaling, act as binding receptors for exosomes to deliver their bioactive cargo to targeted cellular recipients. It has been found that, depending on their origin, exosomes can possess surface proteins that initiate immunomodulatory effects, showing promise in maintaining chronic inflammatory responses [137,138]. 

**Figure 5:**
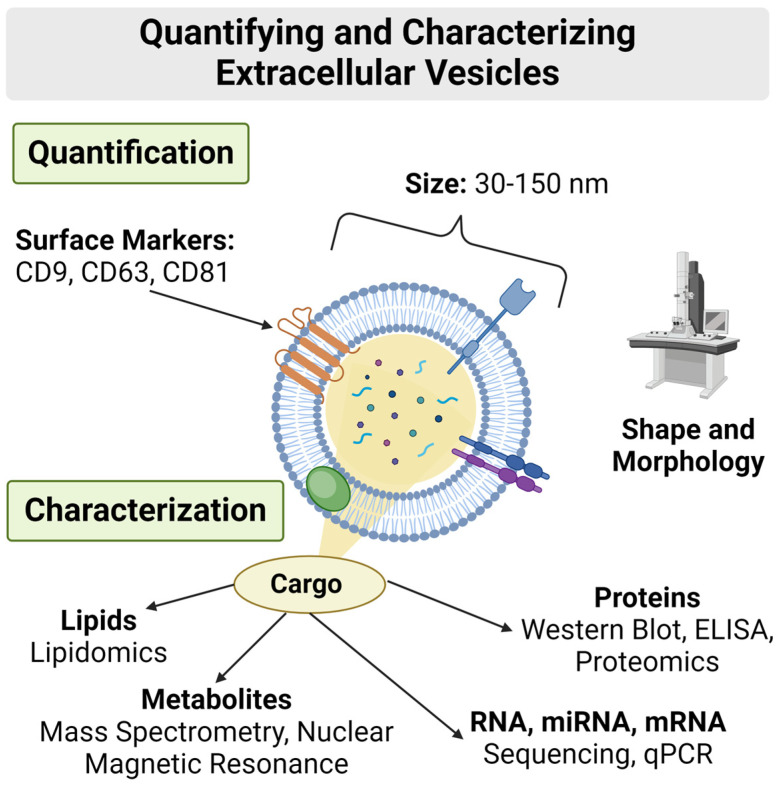
Quantifying and characterizing EVs is an important aspect in identifying their origin and therapeutic potential. Quantification includes analyzing surface markers, particle sizes, particle shapes, and morphology, which is pertinent to differentiating exosomes from their microvesicle counterparts. Characterization of EV cargo furthers our understanding of (1) the environmental stimuli that influences the bioactive cargo from the signaling cell and (2) the signaling expression or suppression in the target cell influenced by the EVs. Created with Biorender.

The cellular and metabolic state origin of an exosome has profound effects on its diverse bioactive cargo. Influenced primarily by external stimuli, the endosomal sorting and generation of MVBs pack exosomes with cargo intended to trigger a signaling cascade [135]. Toxicants can alter the folding of proteins and microRNA (miRNA), altering the microenvironment of recipient cells. If exosomal cargo are affected by stimuli that cause pathological processes to be more active, then finding ways to mediate these processes would be a key area of study. Bioactive cargo commonly found in exosomes include intracellular proteins, miRNA, mRNA, lipids, and metabolites. Exosomes hold the key to shaping the functions of recipient cells, exerting their influence through a plethora of mechanisms including RNA-mediated instructions, the generation of cellular machinery messenger RNA (mRNA), and the regulation of biochemical processes and cellular activation [125,139]. Recently, researchers have been utilizing the presence of known RNA subsets in exosomes—a diagnostic tool to help understand disease progression better [140]. Understanding these mechanisms is a crucial aspect of understanding the impact exosomes have in regulating and manipulating cellular environments (Figure 5). The ultimate objective is to gain precise control over exosome contents, paving the way for their optimization as versatile therapeutic or drug delivery devices, marking a pivotal chapter in biomedical innovation [141]. 

#### 3.4.4. Targeting and Delivery

Exosomes are versatile cargo carriers and active participants in cellular communication, capable of interacting with cells throughout the body, triggering signaling and cellular events. Exosomes deliver their cargo with their target cells through membrane-bound receptors, direct membrane fusion, and endocytosis [142,143]. The structural characteristic of exosomes endows them with the ability to fuse directly with target cells, delivering their payload and ensuring the transfer of vital molecular information [136]. Surface proteins embellish exosomes and play a multifaceted role in cellular interactions. Some of these proteins engage in receptor–ligand interactions which can initiate a chain reaction, triggering intracellular signaling cascades that modulate various cellular responses. Endocytosis is a cellular process involving exosome engulfment by cellular membranes [131,144]. Once internalized, exosomes travel to lysosomes, where they face degradation, and their cargo is repurposed or discarded [90]. In some intriguing cases, exosomes may undergo modification within the endosomal pathway, acquiring additional cellular cargo before being re-secreted into the extracellular space (Figure 4). This process, known as exosome recycling, further underscores exosome-mediated communication’s dynamic and intricate nature [145,146]. 

#### 3.4.5. Impact

Expanding pre-clinical research on exosomes has provided a template for developing therapeutics that utilize the unique characteristics of exosomes to enhance the regeneration of damaged tissues [147]. Due to exosomes’ ability to effectively shuttle stimuli-influenced cargo, the role of exosomes in AC damage may be either inducing or suppressive [148,149]. With the ability to utilize exosomes as potential diagnostic biomarkers, identifying the cargo-influenced mechanisms directing these contradictive effects is a key step in manipulating exosome cargo for targeted therapeutic approaches [143,150]. In addition, exosomes can act as drug carriers as they are non-immunogenic, avoid phagocytosis and engulfment by lysosomes, and expand their potential in pharmaceuticals [151,152,153]. Exosomes represent a promising therapeutic tool poised to enhance the effectiveness of existing orthobiologics while offering a safer and more targeted approach to delivering regenerative and immunomodulatory factors [85,154,155,156,157]. As previously stated, they can provide a more targeted and potent method of delivering regenerative and immunomodulatory factors, while reducing the potential risks associated with uncontrolled cellular responses. As versatile cargo, exosomes offer a unique opportunity to utilize therapeutic agents by encapsulating them within scaffolds or hydrogels as a new strategy for drug delivery in cartilage repair. These scaffolds can have a combined therapeutic effect by releasing specific drugs such as BMP with exosomes to enhance cartilage repair [158,159]. This approach allows for the controlled and sustained release of bioactive molecules directly to the site of injury [160]. Encapsulating exosomes in a localized manner can efficiently promote chondrocyte production while minimizing systemic side effects [161]. 

#### 3.4.6. PRP-Derived Exosomes

Platelet activation is pivotal in the body’s natural response to tissue injury. Activated platelets release growth factors and signaling molecules, such as exosomes, that signal endogenous stem cells to migrate to the injury site, initiating the tissue repair process [155]. However, the traditional activation of platelets in PRP necessitates the addition of pro-clotting substances such as thrombin or calcium chloride (CaCl_2_) to induce fibrin formation to release platelet-derived growth factor (PDGF), which is important in cell growth and proliferation [29,162]. Recent research has shown that not all PRP products activate the platelets, limiting their potential [163]. PRP-derived exosomes provide an approach to produce more reliable and consistent results in PRP therapy. Liu, Xuchang, et al. compared PRP activation to PRP-derived exosomes, revealing that PRP-derived exosomes may have a better regenerative potential [155]. In addition, this study highlighted the potential for PRP-derived exosomes’ ability to decrease the apoptotic rate of OA chondrocytes, acting as a carrier for the specific growth factors that activate the Wnt/β-catenin signaling pathway [156]. PRP-derived exosomes provide a more reliable and consistent means of delivering regenerative signals to the injury site. This precision in molecular messaging ensures that the recruitment of endogenous stem cells is not dependent on platelet activation.

##### MSC vs. MSC-Derived Exosome Treatment

MSCs are multipotent precursor cells with adipogenic, osteogenic, and chondrogenic potential [164,165,166]. Through the secretion of various cytokines and growth factors, MSCs can promote paracrine anti-inflammatory and trophic effects that promote tissue repair [167]. Numerous studies have compared MSC treatment to treatment with MSC-derived exosomes [108]. It has recently become accepted that the role of MSCs in repair is not a mechanical response, but instead works as an environmental mediator [168]. MSCs may be responsible for stimulating native repair mechanisms and the recruitment of healing cells to the injury site [169]. This new research into MSC exosomes suggests that exosomes mediate the properties of MSCs. Specific exosomes, derived from chosen cell types, might be strategically utilized to modulate inflammatory responses, orchestrate stem cell differentiation, or advance tissue neovascularization. BM-MSCs-derived exosomes have shown promise in harnessing MSC potential by providing better instruction in promoting an environment for native cartilage repair [170,171,172]. 

##### BM-MSC Derived Exosomes Enhance Cartage Repair

The bone marrow microenvironment contains various cellular and structural components that mediate the health and function of resident MSCs [173]. Bone marrow’s cellular and acellular components regulate MSC proliferation, self-regeneration, and differentiations [84]. BMAC is known to promote a pro-regenerative MSC secretome and produce more chondrogenic cells, indicating the potential for cartilage repair [166]. Most studies have focused on the clinical improvement of orthobiologics rather than the regeneration potential. The few studies that have investigated the effect of bone marrow MSCs (BM-MSCs) for cartilage repair have revealed partial restoration of hyaline cartilage with issues of transient fibrocartilage formation and subchondral bone overgrowth [69,174]. These issues show the need to harness the potential of MSCs to communicate the modulating pathways to address these issues specifically. 

Exosomes can potentially enhance the effects of BMAC and the crucial factors MSCs possess [86]. BM-derived exosomes have shown the potential to attenuate the proliferation and migration of chondrocytes. A recent study suggests that BM-MSC-derived exosomes are endocytosed into the chondrocytes, promoting direct communication to promote chondrogenesis into the injury site. Furthermore, BM-MSC-derived exosomes can reduce the effects of IL-1β, a proinflammatory cytokine that drives synovitis and is an inducer of cartilage degeneration, causing OA [175,176,177]. The BM-MSC-derived exosomes’ ability to inhibit IL-1β in any capacity can enhance the proliferation capacity of chondrocytes while increasing their migration to have a more sustained therapeutic effect. Treating cartilage damage with BM-MSC-derived exosomes can lead to the upregulation of COL2A1 protein, while downregulating MMP13 and Runx2, leading to native cartilage formation and ECM synthesis, and inhibiting apoptosis induction [178,179,180,181].

##### mRNA and miRNA Cargo in Exosomes 

mRNA and miRNA play crucial roles in the complex functions of exosomes in cellular communication. mRNA acts as a blueprint for protein synthesis, while miRNA fine-tunes gene expression with precise control. MiRNAs are small non-coding RNA molecules, about 19–24 nucleotides long, serving as post-transcriptional gene regulators [182]. Their primary function is to inhibit the translation of target mRNA into proteins or degrade them, thereby modulating specific protein levels in the cell. MiRNA binds to complementary sequences in mRNA, silencing or optimizing gene expression in various cellular pathways [183]. This regulatory function is vital for maintaining cellular balance, ensuring genes are not overly expressed or suppressed. MiRNAs are involved in many diverse biological processes, from cell differentiation to immune responses.

Moreover, miRNAs carried by exosomes can travel between cells, enabling long-distance communication in the body. This property extends their impact beyond individual cells, allowing for orchestrating biological responses. MiRNA-mediated regulation, facilitated by exosomes, modulates cellular function, ensuring genes are expressed at the right time and in the right amounts to maintain health. This precision underscores miRNAs’ pivotal role in exosome-mediated cellular communication, suggesting their potential as therapeutic targets, especially in cartilage repair [184].

BM-MSC-derived exosome-specific miRNA plays a crucial role in promoting and enhancing chondrogenesis (Table 3). These exosomes navigate the complex processes by selectively modulating miRNA expression, targeting key factors in cartilage regeneration and inflammation, like IL-1β, while suppressing pro-inflammatory factors [185]. It is important to note that unregulated miRNA expression can lead to overexpression of pro-oncogene factors. Managing miRNA concentrations is critical in exosome-mediated therapies to balance the promotion of beneficial cellular responses and to avoid risks associated with unintended oncogene activation [186].

##### Exosomes as a Novel Orthobiologic Direction

**Figure 6:**
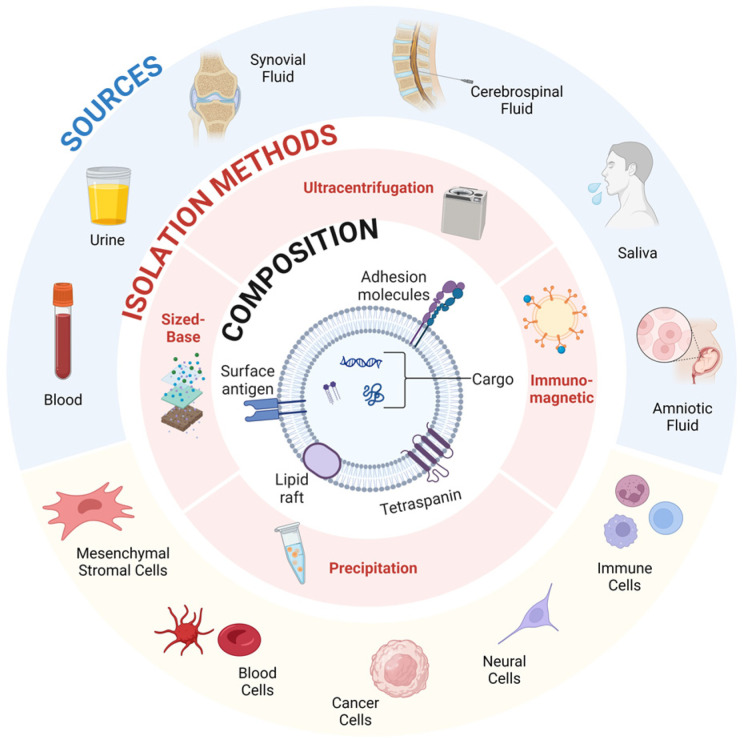
Extracellular overview—EVs are secreted by nearly all cell-types and are found in all bodily fluids and tissues. Common methods of isolating EVs are (1) sized-based filtration, (2) precipitation, (3) immunomagnetic, and (4) ultracentrifugation. Analysis of EV composition provides insight into optimizing recovery efficiency, purity, intact vesicles, and reproducibility. Created with Biorender.

Extracellular Vesicles, such as exosomes, present a novel opportunity to enhance orthobiologics in diagnosing, regenerating, and treating musculoskeletal injuries and diseases. While all cell-types secrete EVs, harnessing beneficial cargo that assists with the homeostasis of the cellular environment presents a strong potential for the targeted cell-sourcing of exosomes. Characterizing and quantifying key components of EV-mediated factors for their use in therapeutic applications has the potential to modulate the cellular environment to homeostasis, promoting healing through EVs promising regenerative and immunoregulatory properties. However, more research is needed to identify the optimal source, optimize isolation, and further characterize exosome-specific bioactive cargo before they are introduced into the clinical space (Figure 6).

## 4. Conclusions

Current therapeutics for cartilage repair have the potential to initiate cartilage regeneration and repair. However, regeneration is limited because of the unique characteristic of articular cartilage which is avascular in nature [14]. Surgical techniques utilize specific ways, such as microfracture, where stem cells in the subchondral bone are stimulated and released within the joint [10,199]. This stimulation process through microfracture leads primarily to the formation of fibrocartilage [21]. Orthobiologics promote the body’s healing response and yield better quality cartilage (hyaline like cartilage) that mimics the native environment [95]. However, orthobiologics such as PRP and BMAC present a similar issue to surgical techniques as there is no clear evidence for regenerating native AC within the joint [200].

Exosome therapy has emerged as an opportunity to direct specific communication within cells without the inherent risks of surgical intervention and the potential for uncontrolled responses to orthobiologics [132]. Exosomes being acellular minimizes the risk of an adverse immune response, and some of the dangers of MSC and stem cell use, such as immune-rejection, can also be minimized [134]. Finally, as exosome production and isolation research continue, approval of an allogeneic exosome product for the treatment of musculoskeletal conditions by the FDA may be more likely than for products with cellular components. Commercial production of an exosome product could lead to an opportunity to increase and control dosing that ensures the best environment for tissue regeneration in the joint.

## 5. Future Perspectives

Tissue engineering is an essential field in regenerative medicine that aims to create functional replacements of damaged or diseased tissue that resembles the native niche [201,202]. It is an emerging interdisciplinary field that uses biomedical techniques combined with current biologics to enhance and precisely regulate the local cellular environment, promoting a sustainable effect for proper tissue regeneration. Biomaterial scaffolds are a specific technique in tissue engineering that utilize a three-dimensional framework that supports the differentiation and proliferation of cells to repair and regenerate new tissue [203,204]. Scaffolds provide a porous structure that closely resembles the ECM found in tissues by utilizing biomaterials to influence biological processes to promote tissue regeneration [205,206]. New acellular scaffold therapies have become an interesting topic in cartilage repair as it offers an opportunity to aid, and one day replace, traditional surgeries such as bone marrow stimulation and ACI surgery.

To be successful in regenerating native tissue and maintaining structural compatibility, acellular scaffolds need to have a similar composition to AC [207]. They need to be constructed with mechanical strength and flexibility to withstand the force load that joints encounter while being biocompatible with the surrounding joint region to mimic and regenerate the existing ECM [208,209]. Supporting cell infiltration is critical for the proliferation and differentiation of chondrogenic and osteogenic cells; using natural and synthetic polymers while loading them with bioactive elements can enhance the cellular components needed for native tissue regeneration [210]. A multilayer and multipolymer approach has been shown to be the most effective acellular scaffold structure. A recent review article described two multilayered acellular scaffolds, MaioRegen and Agili-c, that have published data regarding osteochondral defects, revealing potential cartilage repair [211]. MaioRegen is a trilayered acellular scaffold that is composed of type 1 collagen and hydroxyapatite, while Agili-C is a crystalline aragonite bi-phasic scaffold that is combined with HA; the two unique properties can stimulate the cell infiltration process leading to better quality ECM production [211].

Acellular scaffolds can also be used as a delivery system for bioactive carriers such as MSCs and exosomes [209,212]. The fabrication of acellular scaffolds as a platform for the integration of exosomes has recently been studied, and this has shown that utilizing 3D culture, nanomaterials, and 3D biomaterials can enhance the exosome effect on the joint, leading to cartilage repair [207,213]. In one study, Liu et al. utilized a photoinduced imine crosslinking hydrogel glue as an exosome scaffold for cartilage defect regeneration [214]. This study showed that this “acellular tissue patch” could retain the bone marrow-derived exosomes and regulate chondrocyte production while integrating with native cartilage, resulting in cartilage repair [214]. In addition, an exciting class of biomaterials that are able help regenerate bone and cartilage regrowth is bioceramics, a biocompatible ceramic that has “apatite formation ability, allowing bonding to form between material and tissues” [215]. Bioactive bioceramics are commonly incorporated into scaffolds to provide structural support as they contain materials such as hydroxyapatite, tricalcium phosphate, and bioactive glass [215,216,217]. One group hypothesized that a scaffold based on gelatin/PRGF/lithium-doped bioactive glass seeded by endometrial stem cells forms a useful model for bone regeneration. Reza-Farmani et al. revealed that lithium ion increases cell survival through the activation of the Wnt/β-catenin pathway, and with the addition of PRGF it can increase it even further. In addition, lithium can “stimulate stem cells to secrete exosomes”, revealing the ability to elicit a paracrine effect [216]. The release of ions such as lithium is an essential part of the combinatorial effect that bioceramics can have with therapeutics, and exosomes would be a favorable target as this group showed the paracrine effect released by PRGF that increased cell viability [216,218].

These recent studies lay the groundwork for integrating biologics and biomaterials in order to stimulate healing within injured joints. Providing a dynamic scaffold to support the physical native structure while targeting the cellular environment indicates a novel approach towards regenerating AC. Combining targeted bioactive systems indicates the need for further research and preclinical applications to understand the exact mechanisms of action and their translational viability. Integrating cell signaling factors, such as exosomes, with acellular scaffolds is a new frontier in acellular therapies that have the potential to be translated from the bench to the bedside, filling the gap with current surgical and preventative techniques that avoid leaving patients at risk of worsening conditions, such as OA.

## Figures and Tables

**Table 1:** Pertinent growth factors for cartilage repair and maintenance.

Growth Factor	Importance	Sources
Transforming Growth Factor-β (TGF-β)	TGF-β is essential for autonomous cartilage formation. In the absence of TGF-β, cartilage is not formed. However, it has been demonstrated that downregulation of TGF-β can lead to less fibrotic cartilage repair, suggesting that there is an optimum concentration level.	[20,21]
Bone Morphogenic Protein (BMP)	BMPs are prominent growth factors for regenerating osteochondral tissue. Often expressed throughout the whole chondrogenic process, BMP2 has been shown to improve subchondral bone, while BMP4 has been shown to be superior in hyaline cartilage formation. BMP7 has demonstrated efficacy in promoting cartilage differentiation, proliferation, and retention of ECM. Many BMPs have been studied, all sharing similar roles in cartilage formation and maintenance.	[22,23]
Insulin-Like Growth Factor (IGF)	IGF-1 has been linked to increases in proteoglycan and collagen synthesis, as well as to the reduction of ECM degradation.	[24,25]
Interleukin-1 (IL-1)	IL-1 inflammatory cytokine has been shown to lead to cartilage degradation. IL-1 receptor antagonist reduces proteoglycan breakdown.	[26]
Fibroblast Growth Factor (FGF)	Recombinant human FGF-18 has been shown to stimulate chondrocyte proliferation and SOX-9 expression, as well as a marked decrease in type I collagen expression.	[27]
Vascular Endothelial Growth Factor (VEGF)	Increased levels of VEGF have been correlated to OA progression. Specifically, VEGF appears to be involved in endochondral ossification, osteocyte formation, synovitis, and pain. Anti-VEGF treatments show promise for protecting cartilage from degradation and reducing the progression of OA.	[28]
Platelet-Derived Growth Factor (PDGF)	PDGF is highly expressed in the early stages of wound healing and prevalent in platelets and Platelet Rich Plasma (PRP). PDGF plays a role in chondrocyte proliferation and inhibits the endochondral maturation process.	[29]
Tumor Necrosis Factor-alpha (TNF-α)	Another inflammatory cytokine, TNF-α, plays a role in OA progression. TNF-α inhibition has shown efficacy in reducing the progression of OA.	[30]

**Table 2:** Overview of interventional drugs under investigation for microfracture enhancement therapy.

Interventional Drug	Common Use	Known Benefits	Targeted Use	Sources
Losartan	High blood pressure	- Block TGF-β1 - Decrease Fibrosis - Increase Hyaline cartilage production	- Production of type II collagen and hyaline cartilage	[96]
Avastin	Generic cancer treatment	- Block angiogenesis - Inhibiting VEGF - Increase Hyaline cartilage production	- Production of chondrocytes	[97]
Fisetin	Supplement	- Promote apoptosis in senescent cells	- Elimination of senescent cells	[98]

**Table 3:** Common miRNAs pertinent to orthopedics found in exosomes and their function.

miRNA	Orthopedic Function	Sources
miRNA-1	Promote the growth of cartilage through HDAC4.	[187]
miRNA-31	Factor important for growth and proliferation of chondrocytes, and found to be chondroprotective in OA models.	[188]
miRNA-92a	Promote proliferation of cartilage progenitor cells through PI3K.	[189]
miRNA-92a-3p	Regulates cartilage development and homeostasis through Wnt5a.	[190]
miRNA-95-5p	Regulates cartilage development and homeostasis through HDAC2.	[191]
miRNA-100-5p	Maintains cartilage homeostasis through mTOR.	[192]
miRNA-135b	Promotes chondrocyte proliferation and cartilage repair through SP1.	[193]
miRNA-140-5p	Enhances proliferation and migration of chondrocytes through RALA.	[194]
miRNA-145	Promotes chondrogenesis in periosteal cells.	[195]
miRNA-155-5p	Shown to play a role in cell proliferation and apoptosis. Downregulated in OA.	[196]
miRNA-221	Suppresses pro-inflammatory cytokines.	[195]
miRNA-320	Promote the growth of cartilage through mmp13.	[197]
miRNA-486-5p	Upregulated in OA. Linked to cartilage degradation.	[198]

## Data Availability

No new data were created or analyzed in this study. Data sharing is not applicable to this article.
